# Case report: Anal tuberculosis presenting as an anal fistula

**DOI:** 10.3389/fgstr.2023.1129715

**Published:** 2023-04-11

**Authors:** Muhammad Qasim Chaudhry, Shahab Abid, Naila Kayani

**Affiliations:** Department of Medicine, Aga Khan University, Karachi, Pakistan

**Keywords:** gastrointenstinal, tuberculosis, anal fistula, anal tuberculosis, infection

## Abstract

This is a case of a young gentleman, who presented with complaints of hematochezia, weight loss and fluctuating fever for the past five months. The patient was a known case of Hirschsprung disease and Ulcerative colitis (IUC) and underwent a rectal Duhamel procedure in the past. On examination there was a fistula with an external opening at the anal verge. The clinical suspicion at this point was enterocutaneous fistula, abscess, and incontinence secondary to Hirschsprung disease. Investigations including MRI and sigmoidoscopy were carried out. A tissue from the anorectal junction was taken for histopathology review. Histopathological analysis suggested granulomatous inflammation with collection of epithelioid histiocytes along with caseating necrosis. This was consistent with the diagnosis of anal tuberculosis. The patient was started on a quadruple regimen of anti-tuberculous drugs (ATT). The patient six months into treatment has shown significant clinical improvement.

## Highlights

1. Differential diagnosis of perianal conditions should include chronic infection related to TB.
2. Anal TB mimics noninfectious etiologies such as fistula in ano and Crohn’s disease.
3. Tb should be ruled out in endemic areas before starting immunosuppression for suspected Crohn’s disease.
4. The prognosis of anal TB with the proper drug regimen is excellent with complete remission of the disease.

## Background

Tuberculosis is a prevalent disease around the world, with more than one third of the world population infected by Tuberculosis Bacilli. However, in Pakistan, TB remains an endemic despite BCG vaccine being a part of routine post-natal immunization regime. The country is ranked fifth in the highest burden countries around the globe, with a prevalence, incidence and mortality estimated at 348, 276 and 34 per 100,000 individuals respectively (1, 2). Gastrointestinal tuberculosis forms <1% of the TB burden, with anoperineal TB being an extremely rare find (3, 4). We report a case report of anorectal tuberculosis which presented as an anal fistula- the first reported case from our country.

## Case presentation

We report the case of a young 25 year old gentleman who presented to our clinic with complaints of frequent stools, fecal incontinence, blood in feces, painful defecation and straining since the past five months. The blood was fresh red in color, without clots and remained on the surface of the stool. This was accompanied by fever of variable degree, with a maximum recorded temperature of 102-degree Fahrenheit and lower back pain. The patient reported as having abdominal distention, and right lower back pain, with pus-like discharge.

The patient was a known case of Hirschsprung disease and Ulcerative colitis (history of ulcerative colitis extended to the past 3 years; however, there were no imaging records available to corroborate) and underwent a rectal Duhamel procedure, among ten other surgeries over the years. He reported having three blood transfusions over the past three months because of consistently falling hemoglobin levels, without any exogenous source of bleeding. In review of systems, the patient reported decreased appetite and disturbed sleep with repeated wakening. Our patient reported urinary frequency and burning micturition.

On general physical examination, the patient had clubbing, pallor, and melasma. The abdominal exam revealed a scaphoid abdomen with multiple midline scars, due to prior surgeries. There was a fistula with an external opening at the anal verge.

The clinical suspicion at this point was enterocutaneous fistula, abscess, and incontinence secondary to Hirschsprung disease. There was no evidence of the patient suffering from active IUC in the most recent endoscopic review and no history of taking immunosuppressive medication, as a result the IUC was considered to be in remission and not explored further.

## Investigations

**Figure 1:**
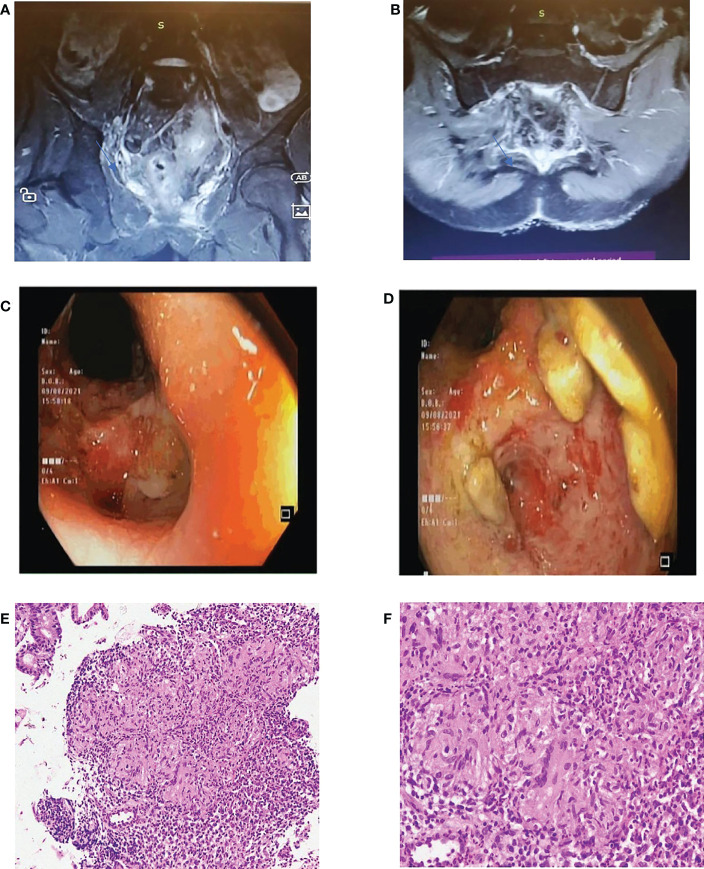
**(A, B)** Coronal and transverse sections of MRI pelvis respectively. **(C, D)**: Sigmoidoscopy images. **(E, F)**: Histopathological analysis of anal biopsy from ulcerated areas with H & E staining- X10 and X100 magnification in E and F respectively.

To evaluate the patient, an MRI pelvis with contrast was carried out. The patient was reported as having mural thickening of the rectum and the rectosigmoid junction, with significant adjacent fat stranding. In the pre-sacral region, the patient has multiple small intercommunicating pockets of collections (8.0 mm x 2.2 mm x 3.2 mm), which were inseparable from the rectosigmoid junction. A linear tract traversing to rectum piercing the internal mucosa at 6 0 clock position with soft tissue enhancement was visualized (**Figures 1A, B**). The MRI was followed by a sigmoidoscopy, which showed severe ulcerations with a friable mucosa. One fistula opening was discovered 3 cm from the anal verge, and another two openings just above the anal verge (**Figures 1C, D**). A tissue from the anorectal junction was taken for histopathology review. (**Figures 1E, F**). PCR was conducted on the collected sample.

Apart from imaging, the patient underwent extensive laboratory workup. Hemogram values included Hemoglobin 15.0 g/dL, white blood cell count 6.5 x10E9/L (monocytes slightly raised at 11.2) and platelets at 534 x10E9/L. ESR and CRP levels were 35mm/hr and 17mg/L respectively. HIV testing done was negative. AFB testing done on the collected tissue sample was negative.

## Differential diagnosis

Histopathological analysis suggested granulomatous inflammation with collection of epithelioid histiocytes along with caseating necrosis (**Figures 1E, F**). This along with the positive PCR test, was consistent with the diagnosis of anal tuberculosis.

## Treatment

The patient after being diagnosed with anal tuberculosis (TB) was started on a quadruple regimen of anti- tuberculous drugs (ATT). The quadruple regimen included Isoniazid. Rifampin, Ethambutol and Pyrazinamide.

## Outcome and follow-up

The patient is still undergoing treatment. However, the patient has reported documented weight gain of 10 kilograms since the start of the treatment. Apart from this he is now able ambulate freely and does not require a wheelchair. Overall, the patient is depicting signs of considerable clinical improvement.

## Discussion

Extrapulmonary TB can infect any viscera of the human body, with or without pulmonary involvement. With a rise in AIDS globally, the incidence of TB has greatly increased. HIV and TB facilitate each other’s progression, with the bacilli releasing growth factors to speed HIV’s infectivity in the host. Similarly, HIV induced immunosuppression can ameliorate the functions of lymphoreticular cells which reactivate dormant mycobacteria. Even though anal lesions are fairly common in AIDS patients, ranging between 16 and 34%, the incidence of anal TB is extremely rare (1, 5).

Literature reports a greater prevalence of Anal TB is male (ratio 4:1) and primarily in the fourth decade of life. This lesion can happen concurrently with pulmonary infection or manifest earlier than the pulmonary lesion. The spread of infection from lungs to the perineum is *via* endogenous sources, usually following an inhalation of respiratory secretions with high loads of bacilli. Even though the former is the primary source of infectivity, other modes of spread are hematogenous, lymphatic from regional lymph nodes and direct extension from neighboring regions (6, 7).

Anal tuberculosis can present with many morphological forms, including ulcerative (most common), verruous, lupoid and miliary. These lesions classically present as superficial ulcerations with hemorrhagic necrotic base and thick mucopurulent discharge (8). The diagnosis of anal TB is challenging and requires histologic analysis. Under the microscope, these lesions resemble an epithelioid and giant cell tubercle, with a caseating necrotic center. Another way of diagnosing anal TB is to do a bacteriological analysis using the Ziehl-Neelsen stain. This diagnosis is further consolidated by evaluating a positive response to anti-tubercular therapy, or by detecting mycobacterial DNA using PCR (9, 10).

The treatment for anal TB is specific four drug antibiotic therapy, and in most regions with multi-drug resistant TB further antibiotics such as Streptomycin are added to the regimen. A surgical intervention is required in cases of bowel obstruction, abscess or tuberculous fistula-in-ano. The prognosis of anal TB, with or without complications, is excellent with complete remission of the disease (4).

High index of clinical suspicion is required to diagnose tuberculosis in an endemic area without any obvious predisposing factors, family history or immunosuppression.

## Data availability statement

The original contributions presented in the study are included in the article/supplementary material. Further inquiries can be directed to the corresponding author.

## Ethics statement

Ethical review and approval was not required for the study on human participants in accordance with the local legislation and institutional requirements. The patients/participants provided their written informed consent to participate in this study. Written informed consent was obtained from the participant/patient(s) for the publication of this case report.

## Author contributions

MC: Wrote the manuscript. SA: Manuscript revision and patient care. NK: Patient care and histopathological analysis. All authors contributed to the article and approved the submitted version.
